# Estimation of polymorphisms in the drug-metabolizing enzyme, cytochrome *P450 2C19* gene in six major ethnicities of Pakistan

**DOI:** 10.1080/21655979.2021.1955809

**Published:** 2021-07-24

**Authors:** Sagheer Ahmed, Saima Gul, Muhammad Akhlaq, Abrar Hussain, Sidrah Tariq Khan, Halimur Rehman, Muhammad Hanif Bangash, Fadwa Al Mughairbi, Muhammad Hamid Hamdard

**Affiliations:** a Department of Basic Medical Sciences, Shifa College of Pharmaceutical Sciences, Shifa Tameer-e-Millat University, Islamabad, Pakistan; b Department of Physical Therapy, Shifa Tameer-e-Millat University, Islamabad Pakistan; c Faculty of Pharmacy, Gomal University, Dera Ismail Khan, Pakistan; d Balochistan University of Information Technology, Engineering and Management Sciences, Pakistan; e Department of Pharmacology, Riphah Institute of Pharmaceutical Sciences, Riphah International University, Islamabad, Pakistan; f Isotopes Production Division, Pakistan Institute of Nuclear Science & Technology, Islamabad, Pakistan; g Department of Psychology, United Arab Emirates University Al Ain United Arab Emirates; h Faculty of Biological Sciences, Kabul University, Kabul Afghanistan

**Keywords:** Pharmacogenetics, cyp2c19 gene polymorphism, pakistan, punjabi, pathan, urdu, seraiki, sindhi, balochi

## Abstract

Interindividual differences in cytochrome P450 (*CYP)* 2C19 activity may result in variations in the therapeutic response to drugs metabolized by this enzyme. Differences at gene level may translate into protein level with consequent impairment of the enzyme activity. As a result patients with such genetic differences might experience undesirable effects or no effect at all. The aim of the present study was to find out the prevalence of allelic and genotype frequencies of low activity variants of CYP2C19 genes in healthy individuals from six distinct ethnicities of Pakistan. Blood sample was taken from healthy volunteers following informed consent. Isolation of the DNA was followed by the PCR amplification and restriction fragment length polymorphism. Selected samples were sequenced by Sanger sequencing. The frequency of major alleles was 84.93% for CYP2C19*2 and 91.85% for CYP2C19*3, while minor allele was present at 15.06% for CYP2C19*2 and 8.14% for CYP2C19*3. For CYP2C19*2, the frequency of *1*1 genotype was 75.80%, *1*2 was 18.27%, and *2*2 was 5.92% whereas for CYP2C19*3, The frequency of *1*1 genotype was 84.19%, *1*3 was 15.30%, and *3*3 was 0.49% in the Pakistani population. A substantial variation in genotype and allelic frequencies was observed in various ethnicities. Our study demonstrates that a significant Pakistani population has at least one minor allele, which indicates a large number of patients potentially being affected by these variations. Especially, a significant genotype frequency of PM suggests implication for the treatment response and severity/frequency of adverse effects in patients receiving drugs metabolized by CYP2C19.

## GRAPHICAL ABSTRACT

**Figure:**
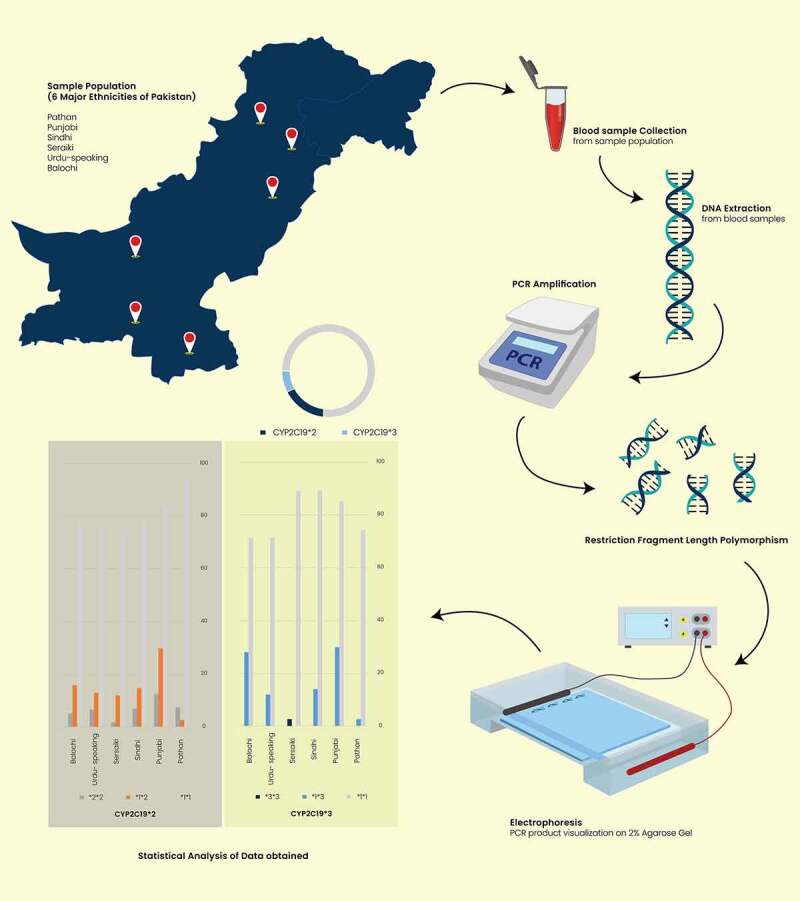

## 1. Introduction

Cytochrome P450 (CYP) is a superfamily of phase 1 enzymes that metabolizes the majority of the clinically available drugs in addition to the synthesis and metabolism of many endogenous substances[1]. This superfamily comprises about 57 enzymes, but only about a dozen carry out the majority of biotransformation reactions and responsible for the metabolism of an overwhelming majority of drugs in the market [2]. Since these enzymes metabolize several different therapeutic agents, polymorphisms in their genes result in variability in their enzymatic activities, which subsequently leads to interindividual variation in the efficacy and adverse effects of the drug [3]. On the other hand, CYP enzymes are also bioengineered to enhance their function such as enhancing steroid hydroxylation efficiency by CYP106A2 [4].

**Figure 1.**
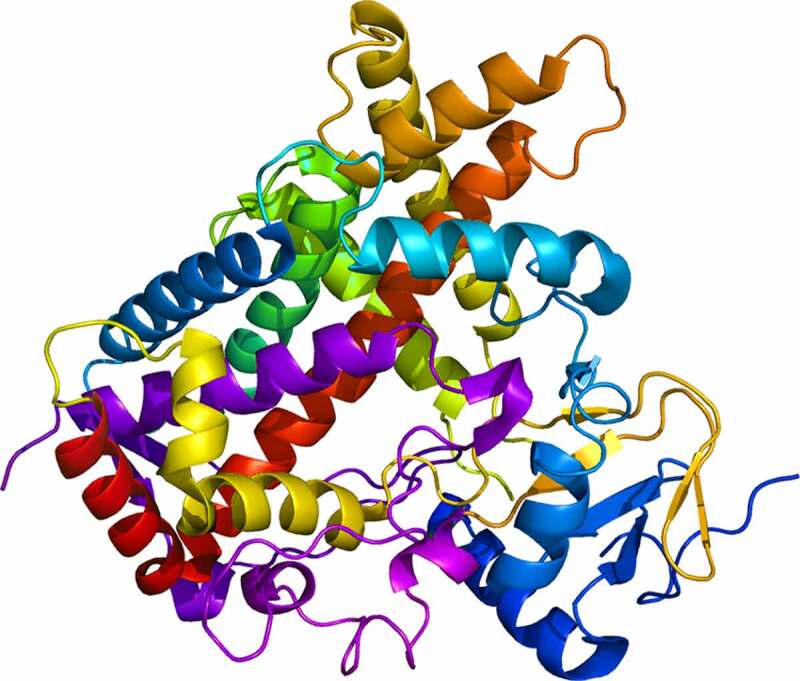
Ribbon struture of CYP2C19 enzyme

CYP2C19 is an important member of the CYP450 superfamily (Figure 1). About one-tenth of all clinically used drugs are metabolized by this enzyme, which includes important classes of medications such as antiepileptics, antidepressants, proton pump inhibitors, antipsychotics, and antiplatelet drugs[1]. Interindividual variation in this gene is likely to yield nonresponder phenomenon or toxic effects in such individuals. The population can be divided into extensive metabolizers (EM) and poor metabolizers (PM) based on whether the enzyme activity is normal or reduced, respectively. Clopidogrel, an antiplatelet drug, is converted to its active metabolite by CYP2C19, and hence PM does not respond to this drug. Therefore, the US FDA has issued a black-label warning for clopidogrel, mentioning its diminished effectiveness in PM [5]. Another prodrug, proguanil, is metabolized to cycloguanil through CYP2C19, but PMs were found to lack cycloguanil, and these patients were at decreased cycloguanil protection and increased risk of Plasmodium infection [6,7].

CYP2C19 enzyme activity is shown to be reduced by more than a dozen alleles, but seven of them produce inactive enzymes. However, two alleles result in the most cases of low activity phenotypes, CYP2C19*2 and CYP2C19*3. CYP2C19*2 is the more common of the two alleles, which leads to the formation of an inactive spliced variant as a result of a G-to-A substitution in exon 5 [8]. CYP2C19*3 is found in exon 4, produces a stop codon with a G-to-A substitution, causing premature termination of transcription and a nonfunctional protein [9]. Both of these alleles provide significant alterations in drug response and adverse effects in PMs. About 50–90% of PM phenotypes can be explained based on these two alleles [10]. The frequencies of these two alleles are higher in the Asian population at 15% compared to Caucasian and African people at 2–5% (Scott et al., 2012). CYP2C19*3 is rarely present in the Caucasian population and is, therefore, considered to be exclusive to the Asian population [3].

Prior knowledge of the frequencies of mutant alleles found in a population may help revise the prescribing practices of the clinicians, including dose adjustments and alternate medications. However, no such data of *CYP2C19* genetic variants are available for the Pakistani population. As mentioned previously, genetic variation in the *CYP2C19* gene affect drugs safety and efficacy. Our hypothesis is that if there is enough generic variation in the *CYP2C19* gene in the context of Pakistani population to warrant further genotype-phenotype kinds of clinical studies. If this variation is low in Pakistani population, then there is not much incentive to carry out further clinical studies. But if the genetic variants that affect drugs safety and efficacy are present in high frequency in Pakistani population, then further studies would be useful as their outcome would affect a sizable fraction of Pakistani population. Therefore, in the current study, our aim was to find out the prevalence of CYP2C19*2 and *3 gene variants in the Pakistani population. We specifically investigated the samples drawn from the six major ethnic groups residing in Pakistan to determine the prevalence of wildtype CYP2C19*1, and CYP2C19*2, and CYP2C19*3 gene variants. One of our goals was to compare the frequencies of the variant alleles observed in the current study, with the global and regional populations.

## 2. Materials and methods

### 2.1 Ethical compliance

This study was conducted at the College of Pharmaceutical Sciences, Shifa Tameer-e-Millat University Islamabad, Pakistan. The study was approved through an approval letter by the institutional review board (IRB#699-147-2016). All participating volunteers were briefed about the study and written informed consent obtained prior to commencing the study. The study was carried out as per the World Medical Association Declaration of Helsinki guidelines.

### 2.2 Sampling & DNA extraction

Samples for this study were collected from various cities of Pakistan. A total of 405 samples were obtained from healthy volunteers belonging to Pathan, Sindhi, Punjabi, Balochi, Urdu-speaking, and Seraiki ethnic groups residing in Pakistan. Ethnicity was self-reprted by the participants. EDTA containing vaccutainers were used to collect approximately 5 ml blood from the each volunteer. Gene Jet Genomic DNA extraction kit was used to extract DNA form the blood. The quality and quantity of the isolated DNA was determined through spectrophotometer and by running it on the agarose gel.

### 2.3 Genotyping

CYP2C19*2 genotyping was done through PCR followed by restriction fragment length polymorphism. An oligonucleotide pair was used which when bound to their complementary sequences on the genomic DNA, would encompass the CYP2C19*2 site. PCR cycles consists of initial denaturation of 10 minutes at 95°C, followed by 35 cycles of denaturation, anealing, and extension, which was followed by a 7-minutes extension at 72°C. The final PCR product was visualized by running it on a 2% agarose gel. After restriction fragment length polymorphism, the three CYP2C19*2 genotypes were identified by running the digested DNA on the 2% agarose gel again.

Similarly, CYP2C19*3 genotyping was performed by using a separate set of oligonucleotides spanning intron 3 and 4 of the *CYP2C19* gene containing CYP2C19*3 allele site. Thermal conditions of this PCR were similar to the ones used for CYP2C19*2 genotyping. Restriction fragment length polymorphism yeided digested products which were run on the 2% agarose gel to determine the CYP2C19*2 genotyping in each sample. More than 40% of the samples were sequenced to confirm the genotyping results.

### 2.4 Statistical analysis

Data obtained from restriction fragment length polymorphism and sequencing were used to determine alleleic and genotype frequencies of the CYP2C19*2 and CYP2C19*3. These allelic and genotype values are provided along with 95% confidence interval values which were calculated as per the following formula, formula (CI = p± (1.96 x SE), SE = qrt [p(1 – p)/n], p = proportion, n = sample size). Difference between the observed and expected frequencies was calculated by Chi-squared test which was also employed to calculate the deviation as per the Hardy-Weinberg equation.

## 3. Results

Our study is the first one to investigate the genetic polymorphisms in *CYP2C19* gene in the healthy individuals of the six distinct ethnic populations of Pakistan-the world 5^th^ most populous country. Our results show that the minor allele was present at a frequency of 15.06% for CYP2C19*2 and 8.14% for CYP2C19*3. Our study shows that a significant fraction of the Pakistani population, and its certain ethnic segments possess high frequency of reduced function polymorphisms. These findings have safety and efficacy implications for our population and encourage further genotype-phenotype studies in hospital and clinical settings involving individual drugs/diseases.

### 3.1 *Allelic and genotype frequencies of* CYPC19*2

**Figure 2.**
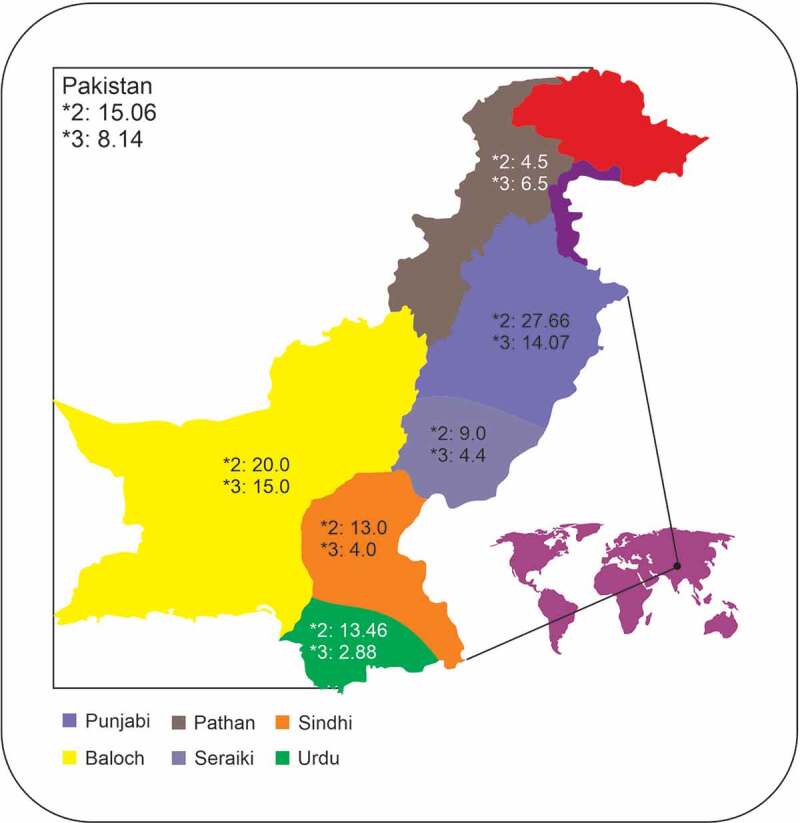
Geographical representation of the ethnicities investigated in the study and their respective CYP2C19*2, and *3 allelic frequencies. The figure was created using CorelDRAW Graphic Suite 2020 (https://www.coreldraw.com/en/)

**Figure 3.**
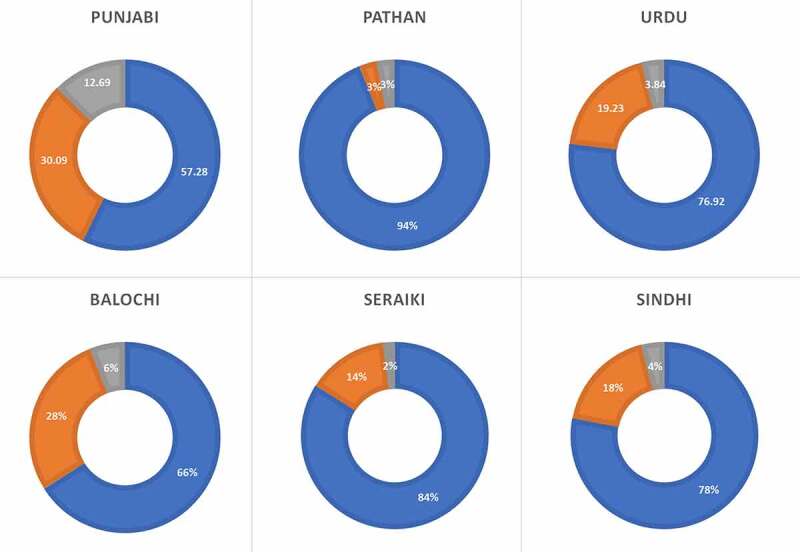
CYP2C19*2 genotype frequencies in various ethnic groups of Pakistan

The prevalence of the CYPC19*2 allele in the various ethnicities of Pakistani population are depicted in Table 1 and Figure 2. The minor allele was found at a frequency of 15.06% in the Pakistani population (Table 1). The Punjabi population exhibited the highest prevalence of the CYPC19*2 followed by Balochi, Urdu, and Sindhi populations. The lowest prevalence of the CYPC19*2 was found in the Pathan population. In the Pakistani population, the prevalence of *1*1 genotype was 75.80%, *1*2 was 18.27%, and *2*2 was 5.92% . The Pathan ethnicity displayed the highest frequency of the wild type genotype (*1*1) at 94%, followed by Seraiki (84%), Sindhi (78%), and Urdu speaking population (76.92%). The Punjabi ethnicity was found to have the lowest wild type genotype frequency (Supplementary Table 1 and Figure 3). However, the Punjabi ethnicity also showed the highest prevalence of heterozygous (*1*2) and homozygous *2*2 genotypes at 30.09% and 12.62%, respectively. The Seraiki ethnicity exhibited th elowest frequency of *2*2 genotype (2%) while the Pathan ethnicity showed the lowest heterozygous genotype (3%).

**Table 1.** Allelic frequencies of CYP2C19*2 allele along with confidence intervals (CI) in various ethnic groups in the Pakistani population

Ethnicity	No.	*1 (CI)	*2 (CI)	Chi-squared Value	p value
**Pakistan**	405	84.93 (82.3–87.3)	15.06 (12.7–17.7)		
**Punjabi**	103	72.33 (65.7–78.3)	27.66 (21.7–34.3)	5.0067	0.0252
**Pathan**	100	95.5 (91.6–97.9)	4.5 (2.1–8.4)	5.5556	0.0184
**Sindhi**	50	87 (78.8–92.9)	13 (7.1–21.2)	0.1661	0.6835
**Balochi**	50	80 (70.8–87.3)	20 (12.7–29.2)	0.8658	0.3521
**Seraiki**	50	91 (83.6–95.8)	9 (04.2–16.4)	1.7045	0.1916
**Urdu**	52	86.53 (78.4–92.4)	13.46 (7.6–21.6)	0.1661	0.6835

### 3.2 *Allelic and genotype frequencies of* CYPC19*3

**Figure 4.**
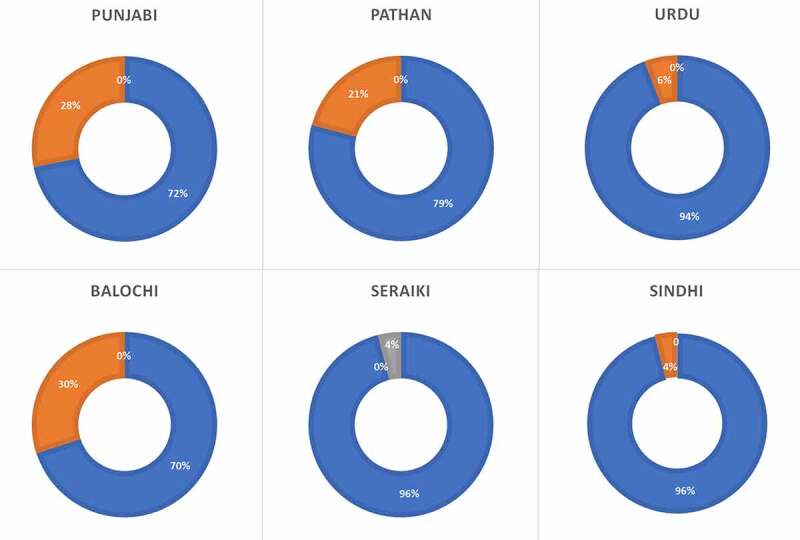
CYP2C19*3 genotype frequencies in various ethnic groups of Pakistan

Frequencies of the CYPC19*3 allele in the Pakistani population are shown in Table 2. This variant was found at a lower frequency than the CYPC19*2 (8.14% vs. 15.06%). The Punjabi population displayed a higher prevalence of this polymorphism compared to other ethnicities, although the highest frequency of the allele was found in Balochi ethnicity (15%). In the Pakistani population, the prevalence of *1*1 genotype was 84.19%, *1*3 was 15.30%, and *3*3 was 0.49%. The highest frequency of *1*1 genotype was found in the Sindhi and Seraiki population at 96% (Supplementary Table 2 and Figure 4). The highest frequency of heterozygous genotype (*1*3) was found in the Balochi ethnic group at 30%. Homozygous genotype (*3*3) was observed only in the Seraiki population, which was the only population that did not display any heterozygous genotype. No other ethnic groups displayed a *3*3 genotype in the Pakistani population (Figure 3).

**Table 2.** Allelic frequencies of CYP2C19*3 allele along with confidence intervals (CI) in various ethnic groups in the Pakistani population

Ethnicity	No.	% A	% C	Chi-squared value	p value
**Pakistan**	405	91.85 (89.19–94.51)	8.14 (5.48–10.8)		
**Punjabi**	103	85.92 (79.2–92.64)	14.07 (7.35–20.79)	1.8386	0.1751
**Pathan**	100	93.5 (88.67–98.33)	6.5 (1.67–11.33)	0.3072	0.5793
**Sindhi**	50	96 (90.57–100)	4 (0–9.43)	1.4184	0.2336
**Balochi**	50	85 (75.1–94.9)	15 (5.1–24.9)	2.4073	0.1207
**Seraiki**	50	96 (90.57–100)	4 (0–9.43)	1.4184	0.2336
**Urdu**	52	97.11 (92.56–100)	2.88 (0–7.43)	2.4050	0.1209

### 3.3 Comparisons with global data

Tables 3 and 4 show the comparisons of alleleic frequencies of CYP2C19*2 and *3 respectively, in the Pakistani population with worldwide populations. CYP2C19*2 allelic frequencies observed in the present study were different from the Peruvian, Han Chinese, Finnish and various South Asian populations but similar to the Colombian, Mexican, Puerto Rican, Europeans from Utah,, British, Spanish, and Italian populations, as reported in the 1000 Genome Project [11] (Table 3). CYP2C19*3 is not found in European and South American populations but the alleleic frequencies foound in the Hans Chinese were similar to Pakistani freequencies but various South Asian populations displayed significantly different prevalence of this allele (Table 4).

**Table 3.** Comparison of CYP2C19*2 allelic frequencies observed in Pakistan with other populations

Population	CYP2C19*1	CYP2C19*2	Chi-squared	p value
CLM	168	20	2.4464	0.117794
MXL	112	16	0.5781	0.447062
PEL	160	10	10.1582	0.001437
PUR	181	27	0.5737	0.448828
CHS	136	74	43.7328	0.00001
CEU	172	26	0.4733	0.491465
FIN	155	43	5.1482	0.023271
GBR	156	26	0.0705	0.790607
IBS	183	31	0.0442	0.833571
TSI	194	20	4.6304	0.031411
BEB	116	56	29.2649	0.00001
GIH	138	68	34.9771	0.00001
ITU	128	76	51.0783	0.00001
PJL	126	66	37.9825	0.00001
STU	120	84	68.6509	0.00001

CLM-Colombian in Medellin, Colombia, MXL-Mexican ancestory in Los Angeles, California, PEL-Peruvian in Lima, Peru, PUR-Puerto Rican in Puerto Rico, CHS-Southern Han Chinese, China, CEU-Utah residents with northern and western European ancestory, FIN-Finnish in Finland, GBR-British in England and Scotland, IBS-Iberian population in Spain, TSI-Toscani in Italy, BEB-Bengali in Bangladesh, GIH-Gujrati Indian in Houston, TX, ITU-Indian Telgu in UK, PJL-Punjabi in Lahore, Pakistan, STU-Sri Lankan Tamil in the UK.

**Table 4.** Comparison of CYP2C19*3 allelic frequencies observed in Pakistan with other populations

Population	CYP2C19*1	CYP2C19*3	Chi-squared	p value
CLM	188	0	-	-
MXL	128	0	-	-
PEL	170	0	-	-
PUR	208	0	-	-
CHS	200	10	2.773	0.095864
CEU	198	0	-	-
FIN	198	0	-	-
GBR	182	0	-	-
IBS	214	0	-	-
TSI	214	0	-	-
BEB	168	4	7.2654	0.007029
GIH	205	1	15.6556	0.000076
ITU	203	1	15.4865	0.000083
PJL	189	3	10.4983	0.001195
STU	201	3	11.458	0.000712

CLM-Colombian in Medellin, Colombia, MXL-Mexican ancestory in Los Angeles, California, PEL-Peruvian in Lima, Peru, PUR-Puerto Rican in Puerto Rico, CHS-Southern Han Chinese, China, CEU-Utah residents with northern and western European ancestory, FIN-Finnish in Finland, GBR-British in England and Scotland, IBS-Iberian population in Spain, TSI-Toscani in Italy, BEB-Bengali in Bangladesh, GIH-Gujrati Indian in Houston, TX, ITU-Indian Telgu in UK, PJL-Punjabi in Lahore, Pakistan, STU-Sri Lankan Tamil in the UK.

## 4. Discussion

Pakistan, as stated by the statistics bureau, ranks 6^th^ in the most populated countries of the world [12]. Out of the total 94% of Pakistan’s population, the Punjabi population takes up 38.78% making it the largest ethnic group in this country, followed by the Pashtun, Sindhi, Siraiki, Urdu-speaking, and Balochi populations at 18.24%, 14.57%, 10.53%, 7.57%, and 3.57%, respectively [13]. Despite being home to a large and diverse society, no significant amount of pharmacogenetics research has been carried out in Pakistani population. This study aims to partially address the above-stated issue by outlining the frequencies in the two most significant genetic variants in the *CYP2C19* gene.

The current study finds the prevalence of the CYP2C19*2 allele at about 15% in the healthy Pakistani particpants which is in a similar range as found in Denmark, Norway, Sweden, Germany, Portugal, Australia, Saudi Arabia, Tanzania, Ethiopia, and Zimbabwe [14–23]. Egypt and Canada previuosly showed the lowest prevalence of the minor allele at 11% [3,24]. Compared to the Pakistani population, CYP2C19*2 allele was more prevalent in Korean, Chinese Taiwanese, Japanese, North Indian, Venda, African American, and Iranian populations [16–18,25,26] while the Filipino population has the highest prevalence of CYP2C19*2 allele [17]. Among various ethnicities, Punjabi samples revealed the highest frequency of the CYP2C19*2 minor allele follwed by the Balochi ethnicity while the Pathan population showed the lowest prevalence of CYP2C19*2 allele. The lowest prevalence of wildtype genotype was found in the Punjabi ethnicity an dth ehighest in the Pathan population (Figure 3).

The prevalence of the CYP2C19*3 allele in the Pakistani population was about 8.14% and was similar to the one found in the Korean and Japanese populations [17,18]. The Egyptian population exhibited the lowest prevalence of the minor allele [3], while the highest frequency is reported from the Korean population at 11.6%. However, no CYP2C19*3 allele is reported from Canada, Poland, Denmark, Germany, Portugal, Australia, Saudi Arabia, Zimbabwe, North Indian, Venda, and African American. The Baloch ethnic group revealed the highest frequency of the minor allele whereas the lowest prevalence was reported from the Urdu speaking ethnicity. The Sindhi and Seraiki ethnicities displayed the highest wild type genotype whereas the Baloch ethnicity exhibited the lowest wild type genotype. The highest heterozygous genotype is also reported from the Baloch population.

The Pakistani population is a combination of various populations due to the several invasions in the first and second mellenia. Partly owing to the recent British domination of the sub-continet in the 18th to 20th century and the Arab influence from the 8^th^ century, the Pakistani population shows genetic influences from several different populations. The large population size also lends to greater genetic diversity. Several regional populations such as Asian, Middle Eastern and Caucasions blend in the Pakistani population [27]. The genetic structure of various Pakistani ethnic groups has ben elucidated by several internationl human genome consortia through the identification and analysis of multiple gene variants [11,28]. Some studies suggest that the genetic make up of the Pakistani population is akin to both Caucasion as well as South Indian populations [29] while others indicate that these ethnicities are similar to European populations [30,31]. It has been suggested that the contribution of several ethnicities such as Aryan, Arab, Persian, Turkish, Kurdish, Dravidian in the ancestry of the Baloch population is the reason for its extreme divergence from other regional ethnicities seen in the present study [32].

A recent study reported frequencies of CYP2C19*2 polymorphism in various ethnic Pakistani populations and found that CYP2C19*2 is prevalent at 29% [33]. However, their investigation did not include two of the most populous ethnicities, namely Balochis and Urdu speaking. They also did not report the frequencies of CYP2C19*3 polymorphism. In contrast to their study, we confirmed our results by direct Sanger sequencing more than a quarter of our samples. Another recent study reported frequencies of CYP2C19*2 polymorphism in the Pakistani population, comprising 155 healthy individuals from one city of Pakistan. However, about 60% of their study participants belonged to the Urdu-speaking population, while the next highest ethnicity was Sindhi at 6.5%. The frequency of CYP2C19*2 was reported at 31% [34].

Rehman and colleagues reported *CYP2C19* polymorphisms in 100 cardiac patients taking clopidogrel [35]. They reported a very high frequency of CYP2C19*2 polymorphism, but this may be due to possible association of this polymorphisms with cardiac diseases. They also did not report any CYP2C19*3 data. Another investigation reported frequencies of CYP2C19*2 polymorphism in breast cancer patients on triple therapy, including cyclophosphamide, fluorouracil, and doxorubicin. They reported a 27.2% frequency for CYP2C19*2 in 67 breast cancer patients [36]. Another study which was conducted on 527 hypertensive patients and 530 unrelated healthy controls reported a frequency of 75 and 25% of wild type and mutant allele in hypertensive patients, and 64.2 and 35.8% respectively in controls [33]. The frequency of CYP2C19*2 was in a similar range, as was found in our study. The observed difference may be due to the difference in the participants recruited for each study.

## Limitations of the study

Limitations of our study include a relatively low sample size. Sample size could have been increased to get more reliable results. One limitation of our study is that the two SNPs (CYP2C19*2, and *3) were considered independently. Therefore, it is theoreticaly possible that in a heterozygous situation (e.g *1*2), *1 may be *3 instead of *2. This possibility could not be tested in our study. There were no phenotypes (e.g. drug responders, drug concentration sin the blood, etc.) tested in our study. A genotype-phenotype association analysis would have been useful. However, we plan to conduct such studies in the future as we get more resources.

## 5. Conclusions

Based on the information that we have gathered, this study will be the first to report the frequency of CYP2C19*2 and *3 gene polymorphisms amongst the healthy population of several distinct ethnicities of Pakistan. It is known that the CYP2C19 enzyme metabolizes approximately 10% of drugs available in the market [2]. Since the unit doses of the drugs dispensed eah year in the Pakistan is approximately 2.6 billion [37], the CYP2C19 enzyme is expected to metabolize more than 260 million unit doses. Current study demonstrates that a significant Pakistani population has one minor allele (about 30%), which indicates a large number of patients (78 million) potentially being affected by these variations. To further clarify the genotype-phenotype correlation, we recommend further research to be carried out on individual drugs metabolized by the CYP2C19 enzyme. Once a physician knows a patient’s metabolizing status, this information may, presumably, lead to more safe and effective prescribing and better performance of drugs.

## Supplementary Material

Supplemental Material
