# Supplementary material for: Estimation of polymorphisms in the drug-metabolizing enzyme, cytochrome P450 2C19 gene in six major ethnicities of Pakistan
Source: Bioengineered. 2021 Jul 24;12(1):4442–51. doi: 10.1080/21655979.2021.1955809 (PMC8806689; doi:10.1080/21655979.2021.1955809)
Supplement: Supplemental Material [file KBIE_A_1955809_SM9794.zip › suppl/Supplementary table 1clean.docx]

**Supplementary table 1:** Genotype frequencies of *CYP2C19*2* in various ethnic groups in Pakistan.

| **Ethnicity** | **Genotype** | **n** | **Observed % (CI)** | **Expected %** | **Chi-squared value** | **p-value** |
| --- | --- | --- | --- | --- | --- | --- |
| **Pakistani** |  | | | | | |
|  | *1*1 | 307 | 75.80 (71.3- 79.9) | 72.14 | 2.2476 | 0.3250 |
|  | *1*2 | 74 | 18.27 (14.6-22.4) | 25.57 |  | |
|  | *2*2 | 24 | 5.92 (3.8- 8.7) | 2.26 |  |  |
| **Punjabi** |  | | | | | |
|  | *1*1 | 59 | 57.28 (47.2- 67.0) | 52.31 | 2.8484 | 0.2407 |
|  | *1*2 | 31 | 30.09 (21.4- 39.9) | 40.02 |  | |
|  | *2*2 | 13 | 12.62 (6.9- 20.6) | 7.65 |  |  |
| **Pathan** |  | | | | | |
|  | *1*1 | 94 | 94 (87.4- 97.8) | 91.2025 | 3.3214 | 0.1900 |
|  | *1*2 | 3 | 3 (6- 8.5) | 8.595 |  | |
|  | *2*2 | 3 | 3 (6- 8.5) | 0.2025 |  |  |
| **Urdu** |  | | | | | |
|  | *1*1 | 40 | 76.92 (63.2- 87.5 | 74.8906 | 1.0739 | 0.5845 |
|  | *1*2 | 10 | 19.23 (9.6- 32.5) | 23.2889 |  | |
|  | *2*2 | 2 | 3.84 (5- 13.2) | 1.8106 |  |  |
| **Seraiki** |  | | | | | |
|  | *1*1 | 42 | 84 (70.9- 92.8) | 82.81 | 0.4727 | 0.7895 |
|  | *1*2 | 7 | 14 (5.8- 26.7) | 16.38 |  | |
|  | *2*2 | 1 | 2 (1- 10.6) | 0.81 |  |  |
| **Balochi** |  | | | | | |
|  | *1*1 | 33 | 66 (51.2- 78.8) | 64 | 0.6974 | 0.7055 |
|  | *1*2 | 14 | 28 (16.2- 42.5) | 32 |  | |
|  | *2*2 | 3 | 6 (1.3- 16.5) | 4 |  |  |
| **Sindhi** |  | | | | | |
|  | *1*1 | 39 | 78 (64.0- 88.5) | 75.69 | 1.0926 | 0.5790 |
|  | *1*2 | 9 | 18 (8.6- 31.4) | 22.62 |  | |
|  | *2*2 | 2 | 4 (5- 13) | 1.69 |  |  |
