# Supplementary material for: Estimation of polymorphisms in the drug-metabolizing enzyme, cytochrome P450 2C19 gene in six major ethnicities of Pakistan
Source: Bioengineered. 2021 Jul 24;12(1):4442–51. doi: 10.1080/21655979.2021.1955809 (PMC8806689; doi:10.1080/21655979.2021.1955809)
Supplement: Supplemental Material [file KBIE_A_1955809_SM9794.zip › suppl/Supplementary table 2clean.docx]

**Supplementary table 2:** Genotype frequencies of *CYP2C19*3* in various ethnic groups in Pakistan.

| **Ethnicity** | **Genotype** | **n** | **Observed % (CI)** | **Expected %** | **Chi-squared value** | **p-value** |
| --- | --- | --- | --- | --- | --- | --- |
| **Pakistani** |  | | | | | |
|  | *1*1 | 341 | 84.19 (80.64-87.74) | 84.36 | 0.0404 | 0.9800 |
|  | *1*3 | 62 | 15.30 (11.79-18.81) | 14.96 |  | |
|  | *3*3 | 2 | 0.49 (0-1.17) | 0.66 |  |  |
| **Punjabi** |  | | | | | |
|  | *1*1 | 74 | 71.84 (63.15-80.53) | 73.82 | 0.3400 | 0.8436 |
|  | *1*3 | 29 | 28.15 (19.46-36.84) | 24.19 |  | |
|  | *3*3 | 0 | 0 | 2.04 |  |  |
| **Pathan** |  | | | | | |
|  | *1*1 | 87 | 87 (80.41-93.59) | 87 | 0.3391 | 0.8440 |
|  | *1*3 | 13 | 13 (6.41-19.59) | 12 |  | |
|  | *3*3 | 0 | 0 | 1 |  |  |
| **Urdu** |  | | | | | |
|  | *1*1 | 49 | 94.23 (87.89-100) | 94.30 | 0.3387 | 0.8442 |
|  | *1*3 | 3 | 5.76 (0-12.09) | 5.59 |  | |
|  | *3*3 | 0 | 0 | 0.07 |  |  |
| **Seraiki** |  | | | | | |
|  | *1*1 | 48 | 96 (90.57-100) | 92.16 | 6.3481 | 0.0418 |
|  | *1*3 | 0 | 0 | 6.68 |  | |
|  | *3*3 | 2 | 4 (0-9.43) | 0.16 |  |  |
| **Balochi** |  | | | | | |
|  | *1*1 | 35 | 70 (57.3-82.7) | 72.25 | 0.5250 | 0.7690 |
|  | *1*3 | 15 | 30 (17.3-42.7) | 25.50 |  | |
|  | *3*3 | 0 | 0 | 2.25 |  |  |
| **Sindhi** |  | | | | | |
|  | *1*1 | 48 | 96 (90.57-100) | 96.94 | 0.1481 | 0.9286 |
|  | *1*3 | 2 | 4 (0-9.43) | 3.92 |  | |
|  | *3*3 | 0 | 0 | 0.04 |  |  |
